# Prediction of distance visual acuity in presbyopic astigmatic subjects

**DOI:** 10.1038/s41598-021-85313-3

**Published:** 2021-03-26

**Authors:** Rie Hoshikawa, Kazutaka Kamiya, Fusako Fujimura, Nobuyuki Shoji

**Affiliations:** 1 Department of Rehabilitation, Orthoptics and Visual Science Course, School of Allied Health Science, Kitasato University, Sagamihara, Japan; 2 Department of Ophthalmology, School of Medicine, Kitasato University, Sagamihara, Japan; 3 Visual Physiology, School of Allied Health Sciences, Kitasato University, 1-15-1 Kitasato, Minami, Sagamihara, Kanagawa 252-0373 Japan

**Keywords:** Outcomes research, Refractive errors

## Abstract

This study was aimed to determine the effect of the amount of astigmatism on distance visual acuity, and to provide a prediction formula of visual acuity according to astigmatism, in a presbyopic population. We comprised 318 eyes of 318 consecutive patients (158 phakic and 160 pseudophakic subjects) without any eye diseases, except for refractive errors with astigmatism of 3 diopter or less. We assessed the relationship of the spherical equivalent visual acuity (SEVA) with astigmatism, and also provided a regression formula of visual acuity according to astigmatism in such subjects. We found a significant correlation between the SEVA and the amount of astigmatism (r = 0.715, p < 0.001) in the entire study population. We obtained similar results, not only in phakic eyes (r = 0.718, p < 0.001), but also in pseudophakic eyes (r = 0.717, p < 0.001). The regression formula was expressed as follows: y = 0.017x^2^ + 0.125x − 0.116 (R^2^ = 0.544), where y = logMAR SEVA, and x = astigmatism. We also found no significant differences in the SEVA for matched comparison among the with-the-rule (WTR), against-the-rule (ATR), and oblique (OBL) astigmatism subgroups (p = 0.922). These regression formulas may be clinically beneficial not only for estimating the visual prognosis after astigmatic correction, but also for determining the surgical indication of astigmatic correction.

## Introduction

In recent years, the surgical demand for obtaining good quality of vision (QOV) has steadily increased with time, since modern senior subjects have become more active in daily life than before. Therefore, the astigmatic correction plays an essential role in improving the QOV as well as the quality of life, even in a presbyopic population. Indeed, modern cataract surgery has been widely recognized as one of the refractive surgeries to maximize the QOV and subsequent patient satisfaction for such patients.

There have so far been several studies on the effect of astigmatism on visual acuity in phakic and pseudophakic subjects^1–11^. The necessity of astigmatic correction can be influenced by several background factors, such as age, pupil size, higher-order aberrations (HOAs), the status of the eyelid, the axis orientation of astigmatism (with-the-rule (WTR), against-the rule (ATR), and oblique (OBL) astigmatism), the presence of the crystalline lens (phakia vs, pseudophakia), and the type of intraocular lens (IOL) (multifocal IOL vs. monofocal IOL).

It is of importance to grasp the overall relationship of refractive astigmatism with actual visual acuity in a large cohort of astigmatic subjects, in order to establish the estimation method for predicting visual acuity in daily practice. However, there have so far been no established regression formula for predicting visual acuity, according to the amount of astigmatism, in a large cohort of presbyopic population. It may give us basic insights on the visual prognosis after astigmatic correction and the decision-making of the surgical indication of astigmatic correction in such subjects. The purpose of the current study is twofold; to retrospectively assess the relationship between refractive astigmatism and distance visual acuity, and to provide a prediction formula of visual outcomes according to the amount of astigmatism, in a presbyopic population.

## Results

**Figure 1:**
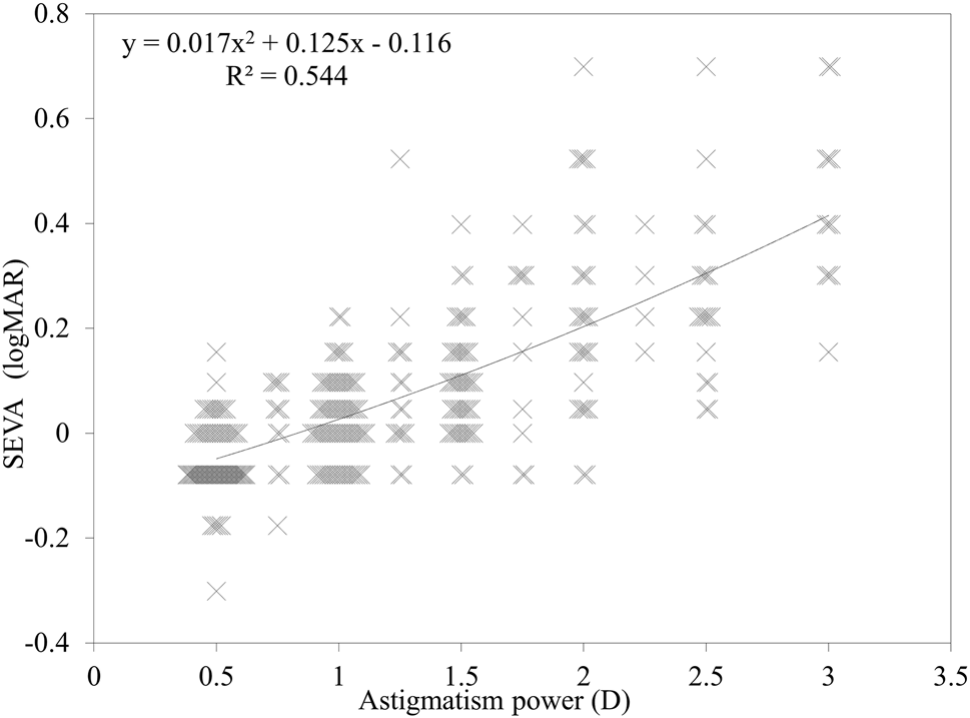
A graph showing a significant correlation between the spherical equivalent visual acuity (SEVA) and the amount of astigmatism in the entire study population (Spearman’s signed-rank test r = 0.715, p < 0.001).

**Figure 2:**
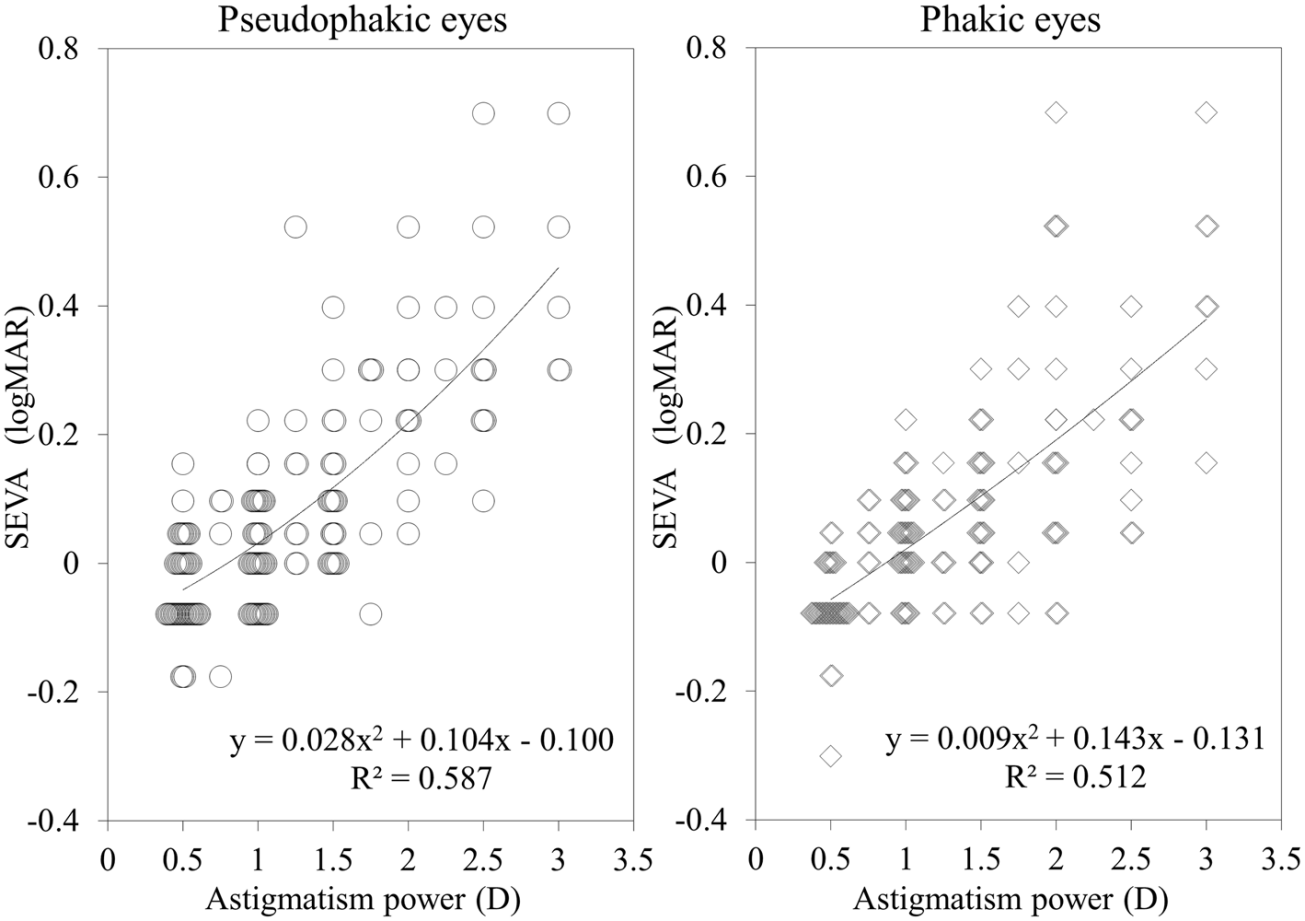
Graphs showing significant correlations between the spherical equivalent visual acuity (SEVA) and the amount of astigmatism in phakic and pseudophakic subgroups (Spearman’s signed-rank test r = 0.718, p < 0.001, and r = 0.717, p < 0.001).

Table 1 shows the demographics of the study population. Figure 1 shows a relationship between the spherical equivalent visual acuity (SEVA) and astigmatism in the entire population. We found a significant correlation between the SEVA and astigmatism (Spearman’s signed-rank test r = 0.715, p < 0.001). The regression formula in the entire population was expressed as follows: y = 0.017x^2^ + 0.125x − 0.116 (R^2^ = 0.544), where y = logMAR SEVA, and x = the amount of astigmatism. After normalization, the regression formula was expressed as follows: y = 0.021x^2^ + 0.103x − 0.102 (R^2^ = 0.629). In a subgroup analysis, we found a significant association between the SEVA and astigmatism, not only in phakic subjects (r = 0.718, p < 0.001), but also in pseudophakic subjects (r = 0.717, p < 0.001) (Fig. 2).

**Table 1:** Demographics of the study population.

Characteristics				P value
	All eyes	Phakic eyes	Pseudophakic eyes	
Number of subjects	318	158	160	
Age	67.6 ± 8.7 years (46 to 89 years)	67.0 ± 8.9 years (49 to 89 years)	68.2 ± 8.4 years (46 to 82 years)	0.070
Gender, male: female	176: 142	93: 65	83: 77	0.210
Astigmatism	1.24 ± 0.68 D (0.50 to 3.0 D)	1.28 ± 0.69 D (0.50 to 3.00 D)	1.20 ± 0.67 D (0.50 to 3.00 D)	0.341
Manifest spherical equivalent	− 1.04 ± 1.99 D (− 6.00 to 2.88 D)	− 0.73 ± 2.34 D (− 6.00 to 2.88 D)	− 1.35 ± 1.52 D (− 5.88 to 1.50 D)	< 0.001
BSCVA	− 0.09 ± 0.04 (0.00 to − 0.30)	− 0.08 ± 0.04 (0.00 to − 0.30)	− 0.09 ± 0.05 (0.00 to − 0.30)	0.127
SEVA	0.07 ± 0.17 (0.70 to − 0.30)	0.07 ± 0.17 (0.70 to − 0.30)	0.08 ± 0.17 (0.70 to − 0.18)	0.688
**Astigmatism**				
WTR	75	24	51	< 0.001
ATR	202	114	88	
OBL	41	20	21	

D = diopter, BSCVA = best spectacle-corrected visual acuity, SEVA = spherical equivalent visual acuity, WTR = with-the-rule, ATR = against-the-rule, OBL = oblique astigmatism.

Table 2 summarizes the outcomes of estimating distance visual acuity according to the amount of astigmatism, based on the above regression formulas: y = 0.028x^2^ + 0.104x − 0.100 (R^2^ = 0.587) for pseudophakic subjects, and y = 0.009x^2^ + 0.143x − 0.131 (R^2^ = 0.512) for phakic subjects, where y = logMAR SEVA, and x = the amount of astigmatism. Phakic subjects tended to show slightly better SEVA than pseudophakic subjects, although there was no significant difference between the two groups (Mann–Whitney U test, p = 0.688).

**Table 2:** Estimated visual acuity according to the amount of astigmatism, based on the regression formulas.

Astigmatism	0.5 D	1.0 D	1.5 D	2.0 D	2.5 D	3.0 D
**Estimated visual acuity (log MAR)**						
All eyes	− 0.05	0.03	0.11	0.20	0.30	0.41
Phakic eyes	− 0.06	0.02	0.10	0.19	0.28	0.38
Pseudophakic eyes	− 0.04	0.03	0.12	0.22	0.34	0.46

**Figure 3:**
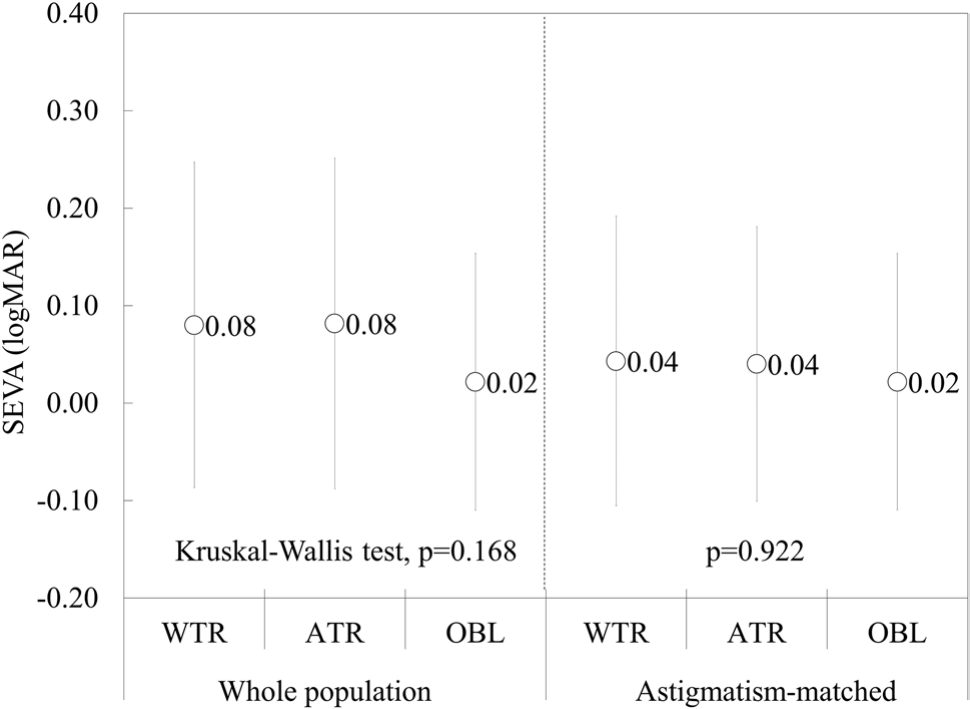
Graphs showing the spherical equivalent visual acuity (SEVA) according to the axis orientation in the entire study population and astigmatism-matched groups. (Kruskal–Wallis test, p = 0.168, and p = 0.922, respectively).

Figure 3 shows the SEVA according to the axis orientation of astigmatism. We found no significant differences in terms of the SEVA among the WTR, ATR, and OBL astigmatic groups (Kruskal–Wallis test, p = 0.168), but the amount of astigmatism was statistically different among the three groups (p = 0.016). When the amount of astigmatism was matched for comparison, we also found no significant differences in the SEVA among the three groups (p = 0.922).

We found a weak, but significant correlation between age and the SEVA (Spearman’s signed-rank test r = 0.201, p < 0.001), and a moderate, significant correlation between astigmatism and the SEVA/the BSCVA (r = 0.662, p < 0.001).

## Discussion

In the current study, our result showed that there was a significant correlation between the amount of astigmatism and the SEVA in a presbyopic population, implying that distance visual acuity significantly worsens, as the amount of astigmatism increases, in such subjects.

The prediction formulas of visual acuity depending on astigmatism were mainly investigated by a non-linear regression analysis^12,13^, except for one study^8^, and thus we have applied a non-linear regression formula in the current study.

Our results may be clinically relevant for understanding that we can usually estimate uncorrected visual acuity of 0.00 logMAR or better even in presbyopic subjects, when refractive astigmatism can be reduced by less than 1.0 D, regardless of the axis orientation, although we accept that pupil size plays a vital role in visual acuity even in astigmatic eyes. We used the SEVA for the assessment of visual performance in astigmatic eyes, when the least circle of confusion is focused on the retina in the current study, in order to precisely investigate the effect of the amount of astigmatism on visual acuity. Remón et al. also evaluated the SEVA when the least circle of confusion is focused on the retina in astigmatic eyes, but the subject age was much younger (25 to 32 years)^10^. To the best of our knowledge, this is the first study to assess the relationship between astigmatism and visual acuity in both phakic and pseudophakic elderly subjects, and to establish a prediction formula of distance visual acuity according to the amount of astigmatism in a large cohort of such subjects. Interestingly, we found some variations in visual acuity, as evidenced by the medium R^2^ value (0.544), even when the amount of astigmatism was identical in the study population. It is suggested that other background factors, such as pupil size, HOAs and the status of the eyelid, might play a role in the variation in the present study^5,7,8,14–16^. With regard to pupil size, Kamiya et al. demonstrated that not only the amount of astigmatism but also pupil size might play an important role in determining visual acuity in young phakic subjects^5^. Watanabe et al. reported that pupil size might have an impact on visual acuity in eyes having ATR astigmatism and in eyes with a large pupil diameter and WTR astigmatism in IOL-implanted eyes^7^. Singh et al. reported that the deterioration in visual acuity with induced astigmatism was lesser for small pupils in pseudophakic subjects, and that the visual acuity loss with induced astigmatism was least with the 1.5-mm pupil diameter, followed by the 3.0-mm, and 6.0-mm pupil diameters^8^. With regard to HOAs, Pujol et al. demonstrated that HOAs reduced the relative loss of retinal image quality introduced by astigmatism^14^. On the other hand, Atchison et al. reported that crossed-cylinder astigmatic blur had a slightly adverse effect on visual acuity than spherical blur in eyes having normal levels of HOAs^15^. We included only eyes with BSCVA of 20/20 or more, and no history of ocular surgery except for cataract surgery. With regard to the status of the eyelid, Sheedy et al. showed that the eyelid squint provides visual benefits in the presence of uncorrected refractive error and overhead glare sources, presumably because of increased depth of focus due to decreased size of the aperture stop^16^. We confirmed that no eyes developed clinically significant ptosis during the visual acuity test. Therefore, we assume that the effect of HOAs and the eyelid on visual acuity was minimum, and clinically negligible, in this study. We believe that this information was simple, but clinically helpful, not only for the surgeons, but also for the patients, to quantitatively grasp the overall tendency of vision, as well as to determine the surgical indication for astigmatic correction, in presbyopic subjects having some astigmatism, although we did not assess other background factors such as pupil size in the study population. In addition, we assessed the relationship between the SEVA and astigmatism after normalization, in order to exclude these background factors as much as possible. In a regression analysis, it is postulated that the data are normally distributed, and that they have equal variances. We used actual astigmatic patients in a clinical setting, and thus the sample size of each astigmatism was mostly different in the current study. The R^2^ value did not substantially increase after normalization in the current study, possibly because the variability of the sample size according to the amount of astigmatism was large. Based on the fact that these R^2^ values are almost identical to that in a previous study using actual astigmatic patients^7^, we believe that it is methodologically acceptable for this kind of clinical investigation.

Table 3 summarizes previous studies on the effect of astigmatism on visual acuity in phakic and pseudophakic subjects. With regard to the amount of astigmatism, visual acuity significantly deteriorated, as the amount of astigmatism increased, in most previous studies^2–11^, which was in line with our findings in a presbyopic population. Hayashi et al. showed that visual acuity at all distances in the monofocal IOL group reduced significantly in proportion to the diopters of astigmatism, and that distance visual acuity in the multifocal IOL group was significantly worse than that in the monofocal IOL group, when astigmatism was 0.5, 1.0, or 1.5 diopters (D)^2^. Watanabe et al. and Yamamoto et al. found a significant association between astigmatism and visual acuity in ATR IOL-implanted eyes, but not in WTR IOL-implanted eyes^7,9^. Yamamoto et al. assessed visual acuity at the second focal line in their study, and mentioned the importance of the locations (the first focal line, the second focal line, and the circle of least confusion) focusing on the retina to assess the UDVA in astigmatic eyes^9^. Remon et al. reported, in a study of 4 young subjects, that the UDVA was better when the focal line is focused on the retina, rather than when the circle of least confusion is focused, but it is still unclear how the UDVA decreased in such conditions^10^. Singh et al. reported, using trial lenses, that the change rate of visual acuity was 0.31 logMAR per D of astigmatism for myopia, and 0.23 logMAR per D for hyperopia, in 15 pseudophakic subjects^8^. By contrast, our findings showed that the change rate of visual acuity was estimated at 0.16 to 0.21 logMAR per D of astigmatism, in a total of 318 phakic or pseudophakic subjects (0.16 to 0.24 logMAR per D of astigmatism in pseudophakic subjects, 0.16 to 0.19 log MAR per D of astigmatism in phakic subjects), and that the overall visual outcomes in the present study was slightly better than those in their previous study. Actually, Ohlendorf et al. showed that visual acuity was worse with simulated astigmatic defocus than real defocus of the same magnitude^17^. The exact etiology of this discrepancy remains unclear, but we assume that the differences in age, pupil size, sample size, focus point, and study design might play a role in this discrepancy.

**Table 3:** Summary of previous studies on the effect of astigmatism on visual acuity in phakic and pseudophakic subjects.

Author	Year	Eyes	Age	Status	Criteria		Focus on retina	Results
					Astigmatism	Refraction		
Trinidade et al.^1^	1997	20	54 to 80	IOL	Actual	Simple myopic astigmatism	Second focal line	WTR = ATR
Hayashi et al.^2^	2000	60	68.9 ± 6.9	IOL	Produced by trial lens	Simple myopic astigmatism	Second focal line	Visual acuity decreased with increasing astigmatism
Remon et al.^3^	2006	4	20	Phakia	Produced by trial lens	Simple myopic astigmatism	Second focal line	Visual acuity decreased with increasing astigmatism
								WTR = ATR = OBL
Wolffsohn et al.^4^	2011	21	58.9 ± 2.8	Phakia	Produced by trial lens	SE 0D	Circle of least confusion	Visual acuity decreased with increasing astigmatism
								ATR > WTR = OBL
Kamiya et al.^5^	2012	20	26.7 ± 4.9	Phakia	Produced by trial lens	Simple myopic astigmatism	Second focal line	Visual acuity decreased with increasing astigmatism and pupil size
Kobashi et al.^6^	2012	38	44.9 ± 19.5	Phakia	Produced by trial lens	Simple myopic astigmatism	Second focal line	Visual acuity decreased with increasing astigmatism
								WTR = ATR > OBL
Watanabe et al.^7^	2013	36	ATR: 68.8 ± 6.8	IOL	Actual	SE + 0.50 to − 0.50D	Circle of least confusion	Visual acuity decreased with increasing astigmatism in ATR eyes
			WTR: 68.3 ± 9.6					WTR = ATR
Singh et al.^8^	2013	15	57.9 ± 9.0	IOL	Produced by trial lens	Simple myopic/hyperopic astigmatism	Focal line	Visual acuity decreased with increasing astigmatism
								WTR = ATR = OBL
Yamamoto et al.^9^	2014	43	ATR: 72.9 ± 7.2	IOL	Actual	Simple myopic astigmatism	Second focal line	Visual acuity decreased with increasing astigmatism in ATR eyes
			WTR: 69.1 ± 8.8					WTR > ATR
Remon et al.^10^	2017	4	25–32	Phakia	Produced by trial lens	Simple myopic/hyperopic astigmatism Compound hyperopic astigmatism Mixed astigmatism	Focal line Circle of least confusion	Visual acuity decreased with increasing astigmatism blur
								WTR = ATR = OBL
Mimouni et al.^11^	2017	17,436	28.1 ± 8.9	Phakia post-LASIK, PRK	Actual	SE + 0.50 to − 0.50D	Circle of least confusion	Visual acuity decreased with increasing astigmatism in ATR eyes
								WTR > ATR = OBL

D = diopter, IOL = intraocular lens, SE = spherical equivalent, WTR = with-the-rule, ATR = against-the-rule, OBL = oblique astigmatism, LASIK = laser in situ keratomileusis, PRK = photorefractive keratectomy.

With regard to the axis orientation of astigmatism, there have been several studies on the relationship between visual acuity and the astigmatic axis, when the circle of least confusion was focused on the retina or close to the retina. Wolffsohn et al. showed that ATR astigmatic eyes showed significantly better visual acuity than the WTR and OBL astigmatic eyes^4^. Watanabe et al. found no significant differences in visual acuity between the WTR and ATR astigmatic eyes^7^. Mimouni et al. reported that the WTR astigmatic eyes showed significantly better visual acuity the ATR and OBL astigmatic eyes, especially in eyes having astigmatism of more than 2.0 D^11^. Yamamoto et al. stated that WTR astigmatic IOL-implanted eyes showed significantly better visual acuity than ATR astigmatic IOL-implanted eyes^9^. Their findings were in line with previous findings^18^, presumably because vertical blur is more tolerable than oblique and horizontal blur. On the other hand, Trinidade et al. demonstrated no significant difference in visual acuity between the WTR and ATR in IOL-implanted eyes^1^. Singh et al. and Remon et al. found no significant difference in visual acuity among the WTR, ATR, and OBL astigmatism produced by trial lenses^8,10^. In the present study, we found no significant differences in visual acuity among the WTR, ATR, and OBL subgroups for the matched comparison. One possible explanation is that the amount of astigmatism is relatively small in the current study. However, since the sample size, the study design, the inclusion criteria, and the methodology were largely different among these previous and current studies, the interpretation of the results should be with caution.

This study has several limitations. First, we did not evaluate pupil size or HOAs in the current study, since our primary goal of this study is to grasp the overall visual performance in elderly population. Second, we did not examine the effect of astigmatism on near visual acuity. Since astigmatism can enlarge the depth of focus, the presence of astigmatism may have advantages over near vision in presbyopic subjects^1,19,20^. A further study on near visual acuity in such presbyopic subjects is necessary to clarify this point. Third, we did not evaluate the impact of neuronal adaptation to astigmatism. It has been shown that human visual system had the adaptation ability to astigmatic defocus^21–23^.

In conclusion, our results showed that there was a significant association between the amount of astigmatism and the SEVA in an elderly population, and no significant difference in visual acuity among the WTR, ATR, and OBL groups. Our results also showed that the predicting formula for visual acuity was made according to the amount of astigmatism in such population. We assume that it will be helpful for estimating the visual prognosis after astigmatic correction, as well as for determining the surgical indication of astigmatic correction in daily practice.

## Materials and methods

### Study population

This study protocol was registered with the University Hospital Medical Information Network Clinical Trial Registry (000040529). This retrospective review of the clinical charts comprised 318 eyes of 318 consecutive subjects (158 phakic subjects and 160 pseudophakic subjects) without any concomitant eye diseases, except for refractive errors. Only one eye for each patient was randomly used for statistical analysis. The inclusion criteria for this study were age ≥ 45 years, BSCVA of 20/20 or more, − 6.0 to + 3.0 D of spherical refraction with astigmatism of 3 D or less, and no history of ocular surgery, except for standard cataract surgery. The exclusion criteria were any concomitant eye diseases such as keratoconus, pellucid marginal degeneration, glaucoma, uveitis, and retinal diseases that could affect visual outcomes, and eyes undergoing toric IOL implantation or multifocal IOL implantation. This retrospective review of the clinical charts was approved by the Institutional Review Board of Kitasato University Hospital (B19-367), and followed the tenets of the Declaration of Helsinki. Our Institutional Review Board waived the requirement for informed consent for this retrospective study.

### Visual acuity measurements

We routinely measured visual acuity using a decimal acuity chart of Landolt rings at 5 m under bright light conditions (500 lx). We used the randomized optotypes in the form of Landolt rings. All Landolt rings were shown in horizontal or vertical direction gaps, and there were 5 Landolt rings in each 1 line between 0.1 and 2.0. The minimum optotype that correctly answered all 5 rings in a row was obtained as a measure of visual acuity. After we obtained objective refractions using an autorefractometer (TONOREF II, Nidek Co. Ltd., Gamagori, Japan), the results were referenced as a starting point for a full manifest refraction. We subjectively determined the astigmatic power and axis using the cross-cylinder method under monocular blind conditions.

We also measured the SEVA, after the correction of the spherical equivalent refraction, calculated as the spherical error + the cylindrical error/2, in order to accurately determine the effect of astigmatism on visual acuity. The SEVA theoretically reflects visual acuity when the least circle of confusion is focused on the retina. Visual acuity was converted to logMAR values, and used for statistical analysis. We assessed the association between age with the SEVA, as well as the relationship of astigmatism with the ratio of the SEVA relative to the BSCVA.

Based on the axis of the steep meridian, we also classified astigmatism as “with-the-rule” (WTR) when the steep meridian was between 60° and 120°, and as “against-the-rule” (ATR) when it was between 0° and 30° or between 150° and 180°. Otherwise, we classified the remaining astigmatism as oblique (OBL) astigmatism.

### Statistical analysis

All statistical analyses were conducted using a statistical software (Bellcurve for Excel, Social Survey Research Information Co, Ltd., Tokyo, Japan). Since all data did not fulfill the criteria for normal distribution by the Shapiro–Wilk test the Spearman correlation coefficient was calculated to assess the relationship between the two variables. The Mann–Whitney U test and the Chi-square test were used to evaluate the differences and the percentages, respectively, between the two groups. The Kruskal–Wallis test was used to compare the data among the three groups. The results were expressed as mean ± standard deviation, and a value of p < 0.05 was considered statistically significant.

### Ethics approval

The study was approved by the Institutional Review Board of Kitasato University and followed the tenets of the Declaration of Helsinki.
